# Achondroplasia and hypochondroplasia in France: a nationwide epidemiological analysis

**DOI:** 10.1186/s13023-025-04069-5

**Published:** 2025-11-03

**Authors:** Genevieve Baujat, Marc-Antoine Hamandjian, Anne-Sophie Jannot, Pierre Karam, Valérie Cormier-Daire

**Affiliations:** 1 Department of Genomic Medicine for Rare Diseases, Reference Center for Skeletal Dysplasia, Necker- Enfants Malades Hospital, Imagine Institute, Paris Cité University, INSERM UMR 1163, Paris, France; 2 Medical Department, BioMarin, Paris, France; 3 Banque Nationale de Données Maladies Rares, DSN-I&D, APHP, Paris, France; 4 PKCS, Ecully, France

**Keywords:** Achondroplasia, Hypochondroplasia, Epidemiology, France, BNDMR, Prevalence

## Abstract

**Background:**

Achondroplasia (ACH) and hypochondroplasia (HCH) are among the most common forms of skeletal dysplasia, caused by gain-of-function variants in the *FGFR3* gene, leading to disproportionate short stature. The birth prevalence of HCH remains poorly defined. In addition, the reported birth prevalence of ACH in Europe and globally may not be applicable to France, given its relatively high rate of pregnancy terminations for medical reasons. This retrospective study provides the first birth prevalence estimates for ACH and HCH in France, using the French National Registry of Rare Diseases (*Banque Nationale de Données Maladies Rares*, BNDMR).

**Results:**

As of January 2024, 766 patients with ACH (ORPHA:15) and 408 with HCH (ORPHA:429) were identified. Most patients were diagnosed and cared for within the network of constitutional bone diseases centers (ACH: 71.3%; HCH: 63.4%). Overall, 85.5% of ACH cases and 57.2% of HCH cases were related to *de novo* genetic variants (*p* < 0.0001). ACH was diagnosed prenatally in 40.8% and at birth in 40.6% of patients, whereas HCH was diagnosed postnatally in 65.7% of cases (*p* < 0.0001). To estimate live birth prevalence, we focused on pediatric patients (0–15 years) born between 2008 and 2023. The mean (range) live birth prevalence was 3.27 per 100,000 for ACH (1.90–4.03) and 1.31 per 100,000 for HCH (0.54–2.08).

**Conclusions:**

This study provides the first nationwide birth prevalence estimates for ACH and HCH in France, leveraging data from BNDMR. ACH is often identified prenatally, whereas HCH is predominantly diagnosed postnatally. The prevalence of HCH may be underestimated due to under-recognition of milder forms. With the emergence of specific therapies for ACH, and for HCH in the near future, strengthening specialized care pathways is critical to ensure equitable access to timely diagnosis and interventions.

**Clinical trial number:**

Not applicable.

**Supplementary Information:**

The online version contains supplementary material available at 10.1186/s13023-025-04069-5.

## Background

Achondroplasia (ACH) and hypochondroplasia (HCH) are among the most common forms of skeletal dysplasia, both caused by gain-of-function pathogenic variants in the fibroblast growth factor receptor 3 *(FGFR3)* gene, which lead to increased FGFR3 signaling. This disrupts chondrogenesis, resulting in disproportionate shortening of the long bones and characteristic craniofacial features [1–5]. Although ACH and HCH follow an autosomal dominant inheritance pattern, most cases are sporadic. Specifically, approximately 80% of ACH cases result from *de novo* mutations (DNMs) in the offspring of unaffected parents [1–3, 6–8]. Both conditions are classified as paternal age effect disorders, as the risk of DNMs increases with advancing paternal age, notably in fathers over 34 years of age compared with those who are younger [6, 7, 9].

Epidemiological estimates of ACH suggest a worldwide birth prevalence of 4.6 per 100,000 live births (95% confidence interval [CI], 3.9–5.4), based on a systematic review and meta-analysis of 52 studies covering births from 1951 to 2015 [3]. In Europe, a population-based study analyzing 11,402,594 births between 1991 and 2015 identified 434 ACH cases, yielding a prevalence of 3.72 per 100,000 births (95% CI, 3.14–4.39) [7]. Specifically, data from a few French regions indicate that ACH prevalence in this country during 1991–2015 ranged from 3.89 per 100,000 births in Auvergne-Rhône-Alpe to 6.11 per 100,000 births in Paris [7]. Regarding the epidemiology of HCH, no studies have been published to determine its prevalence. However, HCH is also considered a relatively common skeletal dysplasia, with a prevalence that may approach that of ACH (1 per 15,000–40,000 live births), with a later diagnosis (around 3 years old) and likely underdiagnosed in some cases [8, 10].

This study presents the first nationwide epidemiological estimates on ACH and HCH in France, utilizing the French National Registry of Rare Diseases (*Banque Nationale de Données Maladies Rares*, BNDMR [https://www.bndmr.fr/*]).* BNDMR is a national database that collects data on individuals diagnosed with rare diseases and monitored in specialized expert centers across France since 2007 [11]. By leveraging this dataset, our study provides valuable insights into the birth prevalence of ACH and HCH in France, addressing the existing gap in epidemiological data, especially in the absence of prior population-based estimates. This is particularly relevant for ACH, as prenatal diagnosis in France is associated with a relatively high rate of pregnancy terminations for medical reasons, including in the third trimester [12].

## Methods

### Data source

BNDMR was established as part of the National Plan for Rare Diseases #2, funded by the French Ministry of Health, to enable the secure collection and centralization of medical data from all patients monitored within the rare disease expert network in France. This is achieved through BaMaRa, an information system deployed in each expert center. Health professionals enter patient data into BaMaRa at diagnosis and during follow-up, followed by a validated quality control process conducted by data managers. After verification, the data of patients who did not oppose to reuse their information are deidentified and consolidated into a centralized clinical data warehouse [13]. Deidentification is ensured through the national rare disease identifier (*Identifiant Maladie Rare*, IdMR), a permanent 20-digit code that maintains patient privacy while enabling tracking across multiple centers. Generated from an exact match of surname, first name, date of birth, and sex, the IdMR ensures accurate patient follow-up and reconciliation of multiple diagnostic records [13].

### Study design and patients

We conducted a retrospective epidemiological study. For the ACH and HCH populations, we included all patients recorded in BNDMR before January 1, 2024, with the ORPHA:15 and ORPHA:429 codes, respectively, who had a confirmed diagnosis (clinical and/or molecular) and had consented to the reuse of their data. It should be noted that all prenatal diagnoses ending in termination of pregnancy are not systematically recorded in BNDMR; therefore, fetal cases were excluded. Data for the present study were extracted on October 3, 2024.

### Ethics

This non-interventional study was approved by the BNDMR scientific committee (BPD-2018-DIAG-008/-002) and registered with the French Data Protection Authority (CNIL: MR004) and the AP-HP local registry (#20230314171333). In accordance with French law, patients were informed about the inclusion of their data in BNDMR and had the opportunity to object to its use for research. Only those who did not object were included in our study.

### Variables of interest

We collected the following variables of interest: date of birth, sex, residential area, inheritance pattern (sporadic or familial), diagnostic status (confirmed by genetic testing or clinically, probable, ongoing, or indeterminate), timing of diagnosis (at birth, postnatal, antenatal, or indeterminate), and follow-up period. Identification of deceased patients was based on death information recorded in BNDMR and an additional matching process with mortality data from the National Institute of Statistics and Economic Studies (*Institut National de la Statistique et des Études Économiques*, INSEE).

### Statistical analysis

The primary outcome is the live birth prevalence estimate of ACH and HCH in France. Due to incomplete coverage of the BNDMR database before 2008, the number of recorded cases may be underestimated, particularly among individuals over 15 years of age (i.e., born before 2008). To address this limitation, live birth prevalence was estimated by determining the mean number of live births with ACH or HCH, divided by the total number of live births in France during the 2008–2023 period. The yearly number of live births in France was provided by INSEE (https://www.insee.fr/fr/statistiques/2381380#*).*

Categorical variables were described as counts and percentages, and continuous variables were described using mean ± standard deviation (SD), median, interquartile range (IQR), and range. Statistical comparisons were performed using chi-square tests for categorical variables, with significance set at *p* < 0.05. Prevalences were expressed as cases per 100,000 births. Missing values were not imputed. Statistical analysis was conducted using Python programming language version 3.8 (Python Software Foundation, Wilmington, DE).

## Results

### Description of the achondroplasia population

**Fig. 1:**
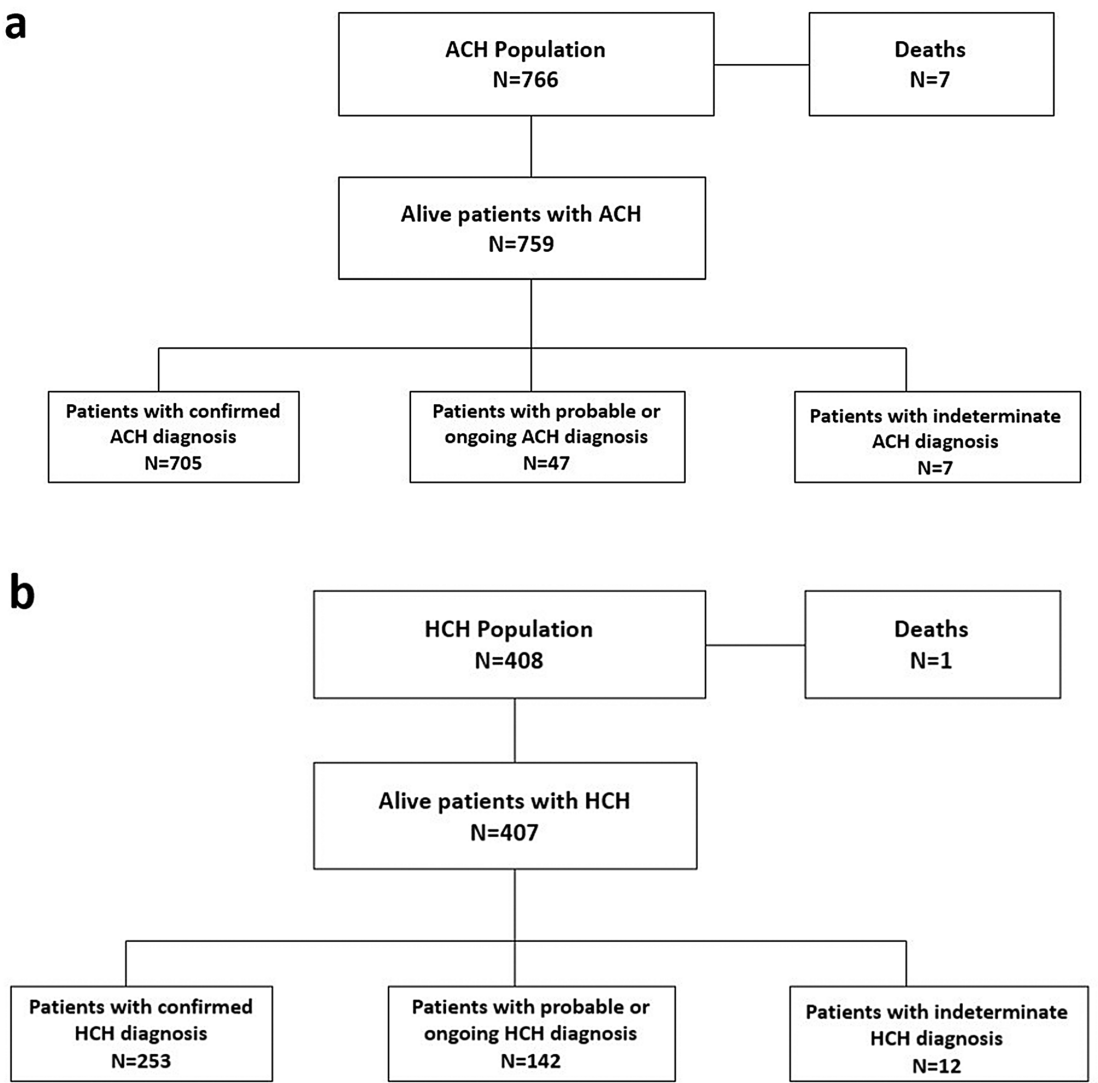
Description of the achondroplasia (ACH) (**a**) and hypochondroplasia (HCH) (**b**) study populations

A total of 766 patients with ACH were included in the French BNDMR database since 2008, among whom 7 (0.9%) had died (Fig. 1a). Most patients (71.3%) were under the care of constitutional bone disease (MOC, *maladies osseuses constitutionnelles*) centers. Table 1 summarizes the demographic and clinical characteristics of the ACH population. The median age in 2024 was 15 years (IQR, 8–28), with a slightly higher proportion of females (55.8%). Overall, 85.5% of ACH cases were related to DNMs (i.e., sporadic inheritance pattern). Among the 598 patients with available data on timing of diagnosis, ACH was diagnosed antenatally in 244 cases (40.8%), at birth in 243 patients (40.6%), and postnatally in 111 patients (18.6%), indicating that approximately 2 out of 5 living patients with ACH were diagnosed *in utero*.

**Table 1:** Demographic and clinical characteristics of included patients with achondroplasia and hypochondroplasia of all ages in France (per BNDMR; up to January 1, 2024)

	Total Population (*N* = 1,174)	ACH patients (*N* = 766)	HCH patients (*N* = 408)	*p*-value
Alive, n	**1166**	**759**	**407**	**-**
Deceased, n	**8**	**7**	**1**	**-**
**Age**,** years**	*N* = 1,163	*N* = 756	*N* = 407	-
Mean ± SD age	19.9 ± 14.7	19.1 ± 14.5	21.5 ± 14.9	-
Median age (IQR) (range)	16 (9–28) (0–85)	15 (8–28) (0–85)	18 (11–27) (0–80)	-
**Sex**,** n (%)**	*N* = 1,163	*N* = 756	*N* = 407	-
Male	528 (45.4)	334 (44.2)	194 (47.7)	0.28
Female	635 (54.6)	422 (55.8)	213 (52.3)	0.28
**Disease inheritance pattern**,** n (%)**	*N* = 1,038	*N* = 664	*N* = 374	-
Sporadic	782 (75.3)	568 (85.5)	214 (57.2)	< 0.0001
Familial	256 (24.7)	96 (14.5)	160 (42.8)	< 0.0001
**Diagnostic status**,** n (%)**	*N* = 1,166	*N* = 759	*N* = 407	
Confirmed (by genetic testing or clinically)	958 (82.2)	705 (92.9)	253 (62.2)	< 0.0001
Probable	120 (10.3)	26 (3.4)	94 (23.1)	< 0.0001
Ongoing	69 (5.9)	21 (2.8)	48 (11.8)	< 0.0001
Indeterminate	19 (1.6)	7 (0.9)	12 (2.9)	0.044
**Timing of diagnosis**,** n (%)**	*N* = 1,064	*N* = 743	*N* = 321	-
At birth	274 (25.8)	243 (32.7)	31 (9.7)	< 0.0001
Postnatal	253 (23.8)	111 (14.9)	142 (44.2)	< 0.0001
Antenatal	287 (27.0)	244 (32.8)	43 (13.4)	< 0.0001
Indeterminate	250 (23.5)	145 (19.5)	105 (32.7)	< 0.0001
**Timing of diagnosis**,** excluding indeterminate**,** n (%)**	*N* = 814	*N* = 598	*N* = 216	-
At birth	274 (33.7)	243 (40.6)	31 (14.4)	< 0.0001
Postnatal	253 (31.1)	111 (18.6)	142 (65.7)	< 0.0001
Antenatal	287 (35.3)	244 (40.8)	43 (19.9)	< 0.0001
**Follow-up period**,** years**	*N* = 1,165	*N* = 759	*N* = 406	-
Mean ± SD duration	9.2 ± 5.5	9.0 ± 5.4	9.7 ± 5.6	-
Median (IQR) duration (range)	9 (5–14) (0–31)	9 (5–14) (0–28)	9 (5–14) (0–31)	-

Abbreviations: ACH, achondroplasia; BNDMR, *Banque Nationale de Données Maladies Rares* [French National Registry of Rare Diseases]; HCH, hypochondroplasia; IQR, interquartile range; SD, standard deviation

### Live birth prevalence of achondroplasia

**Fig. 2:**
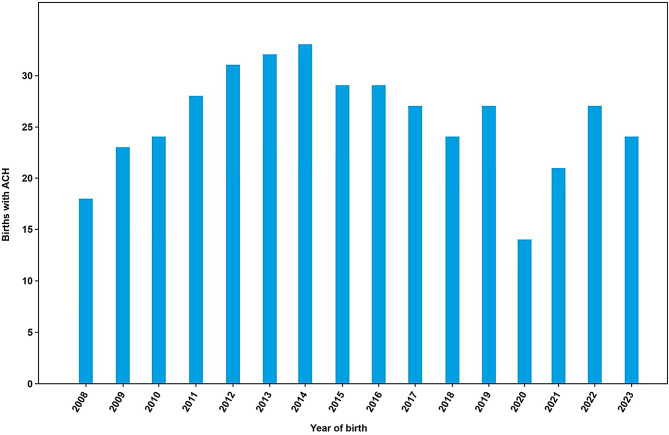
Number of patients with ACH recorded in BNDMR, by year of birth. Abbreviations: ACH, achondroplasia; BNDMR, *Banque Nationale de Données Maladies Rares* [French National Registry of Rare Diseases]

As illustrated in Fig. 2, the number of patients with ACH, by year of birth, ranged from 18 in 2008 to 24 in 2023. According to the total number of live births per year in France from 2008 to 2023 (based on INSEE data), the mean birth prevalence of ACH is 3.27 per 100,000 live births. The birth prevalence of ACH ranged from a minimum of 1.90 per 100,000 live births in 2020 to a maximum of 4.03 per 100,000 live births in 2014 (Table 2).

**Table 2:** Live birth prevalence of achondroplasia in France between 2008 and 2023

Year	Number of live births in France*	Number of live births with ACH^#^	Prevalence of ACH per 100,000 live births
2008	898,404	18	2.00
2009	824,641	23	2.79
2010	832,799	24	2.88
2011	823,394	28	3.40
2012	821,047	31	3.78
2013	811,510	32	3.94
2014	818,565	33	4.03
2015	798,948	29	3.63
2016	783,640	29	3.70
2017	769,553	27	3.51
2018	758,590	24	3.16
2019	753,383	27	3.58
2020	735,196	14	1.90
2021	742,052	21	2.83
2022	725,997	27	3.72
2023	678,000	24	3.54

*As determined by INSEE; ^#^Registered in BNDMR

Abbreviations: ACH, achondroplasia; BNDMR, *Banque Nationale de Données Maladies Rares* [French National Registry of Rare Diseases]; INSEE, *Institut National de la Statistique et des Études Économiques* [National Institute of Statistics and Economic Studies]

Regional prevalence estimates for ACH across France, based on the included population, are presented in Supplementary Table 1. Supplementary Fig. 1 shows the number of patients with ACH by year of birth, distinguishing between those who had at least one recorded visit to an ACH expert within the two years preceding the study cut-off and those who did not.

### Description of the hypochondroplasia population

A total of 408 patients with HCH were included in the French BMDMR database since 2008, among whom 1 patient (0.2%) had died (Fig. 1b). Of them, 63.4% were under the care of constitutional bone disease (MOC) centers. Demographic and clinical characteristics of the HCH population are summarized in Table 1. The median age was 18 years (IQR, 11–27), and the proportion of women was 52.3%. Overall, 57.2% of HCH cases were related to DNMs (i.e., sporadic inheritance pattern), and 42.8% to familial inheritance. Data on timing of diagnosis were available for 216 patients. Among these, HCH was diagnosed antenatally in 43 cases (19.9%), at birth in 31 patients (14.4%), and postnatally in 142 patients (65.7%).

### Live birth prevalence of hypochondroplasia

**Fig. 3:**
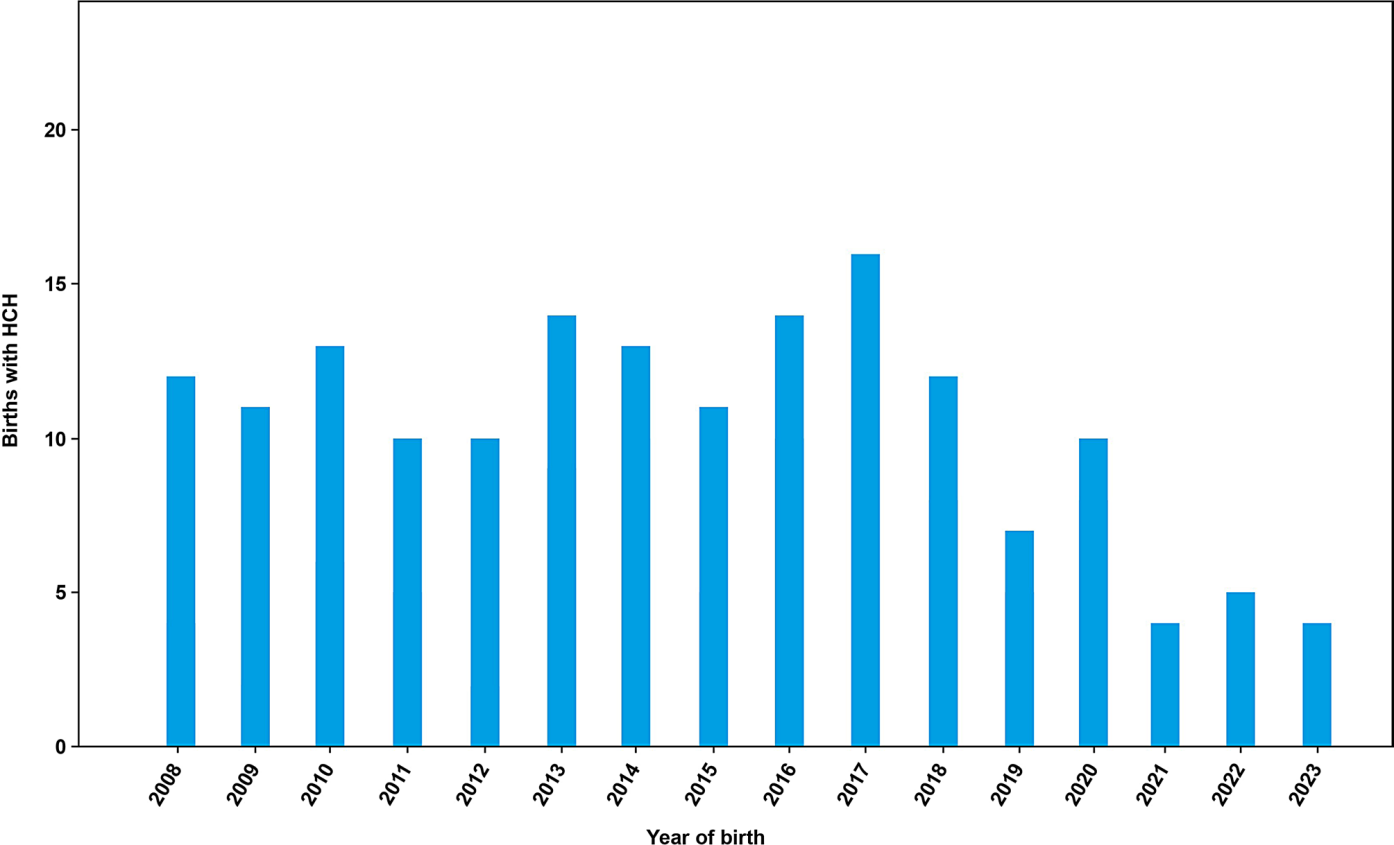
Number of patients with HCH recorded in BNDMR, by year of birth. Abbreviations: BNDMR, *Banque Nationale de Données Maladies Rares* [French National Registry of Rare Diseases]; HCH, hypochondroplasia

The number of patients with HCH by year of birth ranged from 12 in 2008 to 4 cases in 2023 (Fig. 3). According to the total number of live births per year in France from 2008 to 2023 (based on INSEE data), the mean HCH prevalence at birth is 1.31 per 100,000 live births in France, ranging from a minimum of 0.54 per 100,000 in 2021 to a maximum of 2.08 per 100,000 in 2017 (Table 3).

**Table 3:** Live birth prevalence of hypochondroplasia in France between 2008 and 2023

Year	Number of live births in France*	Number of live births with HCH^#^	Prevalence of HCH per 100,000 live births
2008	898,404	12	1.34
2009	824,641	11	1.33
2010	832,799	13	1.56
2011	823,394	10	1.21
2012	821,047	10	1.22
2013	811,510	14	1.73
2014	818,565	13	1.59
2015	798,948	11	1.38
2016	783,640	14	1.79
2017	769,553	16	2.08
2018	758,590	12	1.58
2019	753,383	7	0.93
2020	735,196	10	1.36
2021	742,052	4	0.54
2022	725,997	5	0.69
2023	678,000	4	0.59

*As determined by INSEE; ^#^Registered in BNDMR

Abbreviations: BNDMR, *Banque Nationale de Données Maladies Rares* [French National Registry of Rare Diseases]; HCH, hypochondroplasia; INSEE, *Institut National de la Statistique et des Études Économiques* [National Institute of Statistics and Economic Studies]

Regional prevalence estimates for HCH across France, based on the included population, are presented in Supplementary Table 2. Supplementary Fig. 2 shows the number of included patients with HCH by year of birth, distinguishing between those who had at least one recorded visit to an HCH expert within the two years preceding the study cut-off and those who did not.

## Discussion

This study provides the first national birth prevalence estimates for ACH and HCH in France, using data from BNDMR, a comprehensive database that systematically collects information on individuals diagnosed with rare diseases in specialized expert centers nationwide [11, 13].

Our findings reveal an estimated mean birth prevalence of 3.27 per 100,000 for ACH and 1.31 per 100,000 for HCH, contributing valuable data to the limited epidemiological information on skeletal dysplasias. These results highlight the utility of centralized rare disease registries in capturing population-level disease burden [13]. The prevalence of ACH observed in our study aligns with previous European estimates (3.72 per 100,000) [7] and worldwide prevalence estimates (4.6 per 100,000) [3]. Our study also provides the first population-based estimate of HCH prevalence in France, addressing an important gap in the literature [8, 10].

Despite being recognized as a relatively common form of skeletal dysplasia, the lower birth prevalence of HCH observed in the present study compared to ACH is likely due to the heterogeneity in its clinical presentation. HCH manifests with varying degrees of severity, which may influence whether affected individuals seek medical attention, thereby impacting reported prevalence rates [8, 10]. In newborns with HCH, birth weight and length are often within subnormal ranges, and limb-to-trunk disproportion is typically mild during infancy [8, 10]. Radiological HCH signs are rarely specific and usually moderate during the first few years of life. However, the variable clinical presentation of HCH can contribute to underdiagnosis or delayed diagnosis, potentially leading to a selection bias for HCH in the BNDMR database, as only the more severe cases may have been referred to expert centers [1, 8]. In our study, most of the HCH cases were diagnosed postnatally (65.7%). This is consistent with published reports, as it is well-known that the body height growth curve of people with HCH begins to deviate from the expected height in the general population after the first year of life [8, 10]. These findings highlight the need to increase awareness of HCH among healthcare providers to improve early recognition and timely referral to expert centers. Diagnostic confirmation was also more frequent in ACH cases (92.9%) than in HCH cases (62.2%) (*p* < 0.0001), emphasizing the need for improved access to genetic testing in HCH, particularly given its phenotypic overlap with other skeletal dysplasias [10].

Consistent with prior reports [1, 7], our findings confirm that ACH is predominantly caused by DNMs, with 85.5% of cases classified as sporadic. Although the risk of DNMs increases with advancing paternal age, other potential environmental exposures affecting spermatogenesis have also been hypothesized. Further research is needed to explore whether external factors contribute to the frequency of pathogenic *FGFR3* variants [9], and whether the prevalence of these two conditions is consistent worldwide. In contrast, familial inheritance was more frequently observed in HCH (42.8%) than in ACH (14.5%) (*p* < 0.0001). The higher percentage of familial inheritance in HCH compared to ACH may be attributed to the selection bias of live births, given the more frequent terminations of pregnancy in ACH compared to HCH, as well as potential differences in family planning between parents of individuals with ACH and HCH. Specifically, parents of children with HCH may be possibly more likely to accept offspring than parents of children with ACH [12].

In our study, 40.8% of living individuals with ACH received an antenatal diagnosis, and an additional 40.6% were diagnosed at birth, reinforcing the notion that ACH is often recognized early due to its distinct clinical presentation [1]. However, the prenatal diagnosis rate of ACH (40.8%) found in this database is lower than that reported in previous studies (66.6%) [14]. This discrepancy may be explained by the fact that our study reports the rate of prenatal diagnosis among live births with ACH and does not include prenatally diagnosed cases that resulted in termination of pregnancy. It is likely that the rate of prenatal diagnosis of ACH has increased in recent years thanks to improved screening and diagnostic techniques.

We observed that a notable percentage of pediatric patients did not have at least one follow-up visit recorded with an ACH coordinating expert during the last two years of the study. While this observation appears inconsistent with international and French national recommendations for managing ACH [4, 15–17], it is important to consider potential biases, such as unrecorded follow-up consultations, in either other specialties like otorhinolaryngology, orthopedics, or pulmonology, or within multidisciplinary clinics.

Vosoritide, the first specific treatment authorized for ACH, is indicated to increase linear growth in pediatric patients aged 4 months and above with ACH and open epiphyses. Regular follow-up at specialized centers allows for timely assessment of treatment eligibility and overall care needs [16]. In this context, the general medical community should be reminded of the value of encouraging patients and families to maintain regular consultations with an ACH expert [4]. Patient associations can also play a key role in guiding patients and their families, helping to facilitate access to appropriate care. Finally, health systems must ensure that expert centers have sufficient capacity and resources to meet the growing demand for rare disease care [4].

A limitation that must be acknowledged in the current analyses is that the BNDMR database does not capture an exhaustive population of individuals with ACH and HCH but rather reflects those receiving care in specialized centers across France and who have been seen by a coordinating physician. As such, prevalence may be underestimated, particularly for individuals with the less severe phenotypes who do not seek specialized follow-up. Additionally, the lack of cause-of-death data in the BNDMR database precludes assessment of whether mortality was associated with ACH or HCH. A further limitation is the absence of data on pregnancy terminations following prenatal diagnosis, which prevents estimation of the true pregnancy prevalence of ACH and HCH. However, several methodological strengths enhance the reliability of our findings. The use of BNDMR ensures high-quality data collection, with standardized diagnostic coding and a validated patient identification system [13]. Furthermore, the inclusion of mortality data from INSEE enabled an accurate estimation of current disease live birth prevalence.

## Conclusions

This study provides the first national birth prevalence estimates for ACH and HCH in France, leveraging data from BNDMR. Diagnosis of ACH is often supported by prenatal monitoring and early referral to expert centers. In contrast, HCH is more frequently diagnosed postnatally and may remain underdiagnosed in milder cases. Future efforts should focus on improving early recognition of HCH through increased awareness among healthcare providers and broader access to genetic testing. Moreover, given the availability of targeted therapy for ACH and the anticipated development of treatments for HCH, it is increasingly important to strengthen regular care pathways within expert specialized centers, ensuring timely and equitable access to accurate information and appropriate interventions.

## Electronic supplementary material

Below is the link to the electronic supplementary material.

Supplementary Material 1

## Data Availability

Data supporting the findings of this study are available within the paper and its Supplementary Information.

## Abbreviations

ACH: Achondroplasia
BNDMR: Banque Nationale de Données Maladies Rares (French National Registry of Rare Diseases)
CI: Confidence Interval
DNM: de novo Mutation
FGFR3: Fibroblast Growth Factor Receptor 3
HCH: Hypochondroplasia
IdMR: Identifiant Maladie Rare
INSEE: Institut National de la Statistique et des Études Économiques (National Institute of Statistics and Economic Studies)
IQR: Interquartile Range
MOC: Maladies Osseuses Constitutionnelles
SD: Standard Deviation
