# Supplementary material for: Achondroplasia and hypochondroplasia in France: a nationwide epidemiological analysis
Source: Orphanet J Rare Dis. 2025 Nov 3;20:555. doi: 10.1186/s13023-025-04069-5 (PMC12581505; doi:10.1186/s13023-025-04069-5)
Supplement: Supplementary file 1 — Supplementary Material 1 [file 13023_2025_4069_MOESM1_ESM.docx]

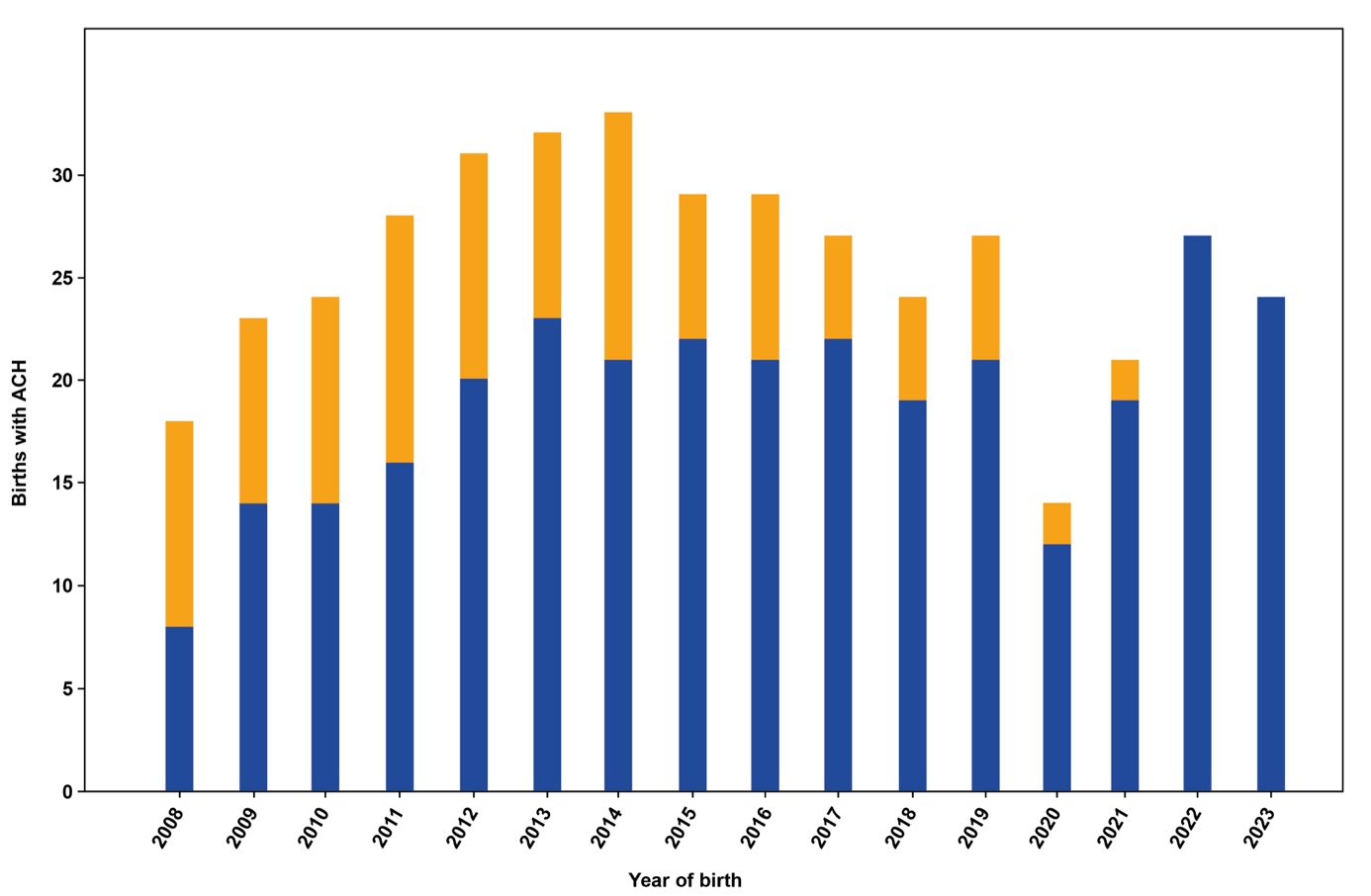


**Supplementary Fig. 1** Number of pediatric patients (0–15 years) with ACH recorded in BNDMR by year of birth, distinguishing those with at least one visit to an ACH expert in the past two years (blue) from those without such a visit (orange). Abbreviations: ACH, achondroplasia; BNDMR, *Banque Nationale de Données Maladies Rares* [French National Registry of Rare Diseases]*.*


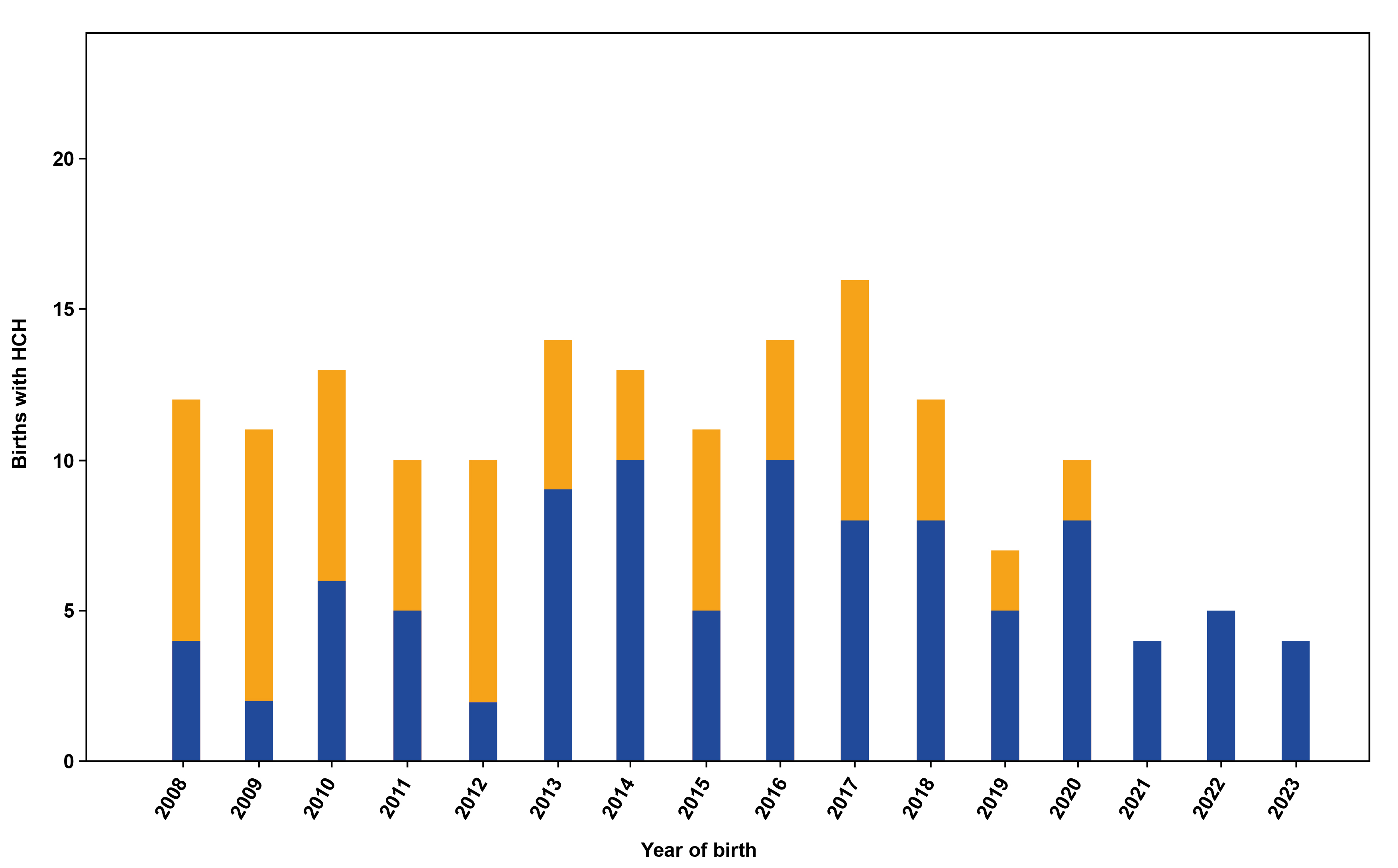


**Supplementary Fig. 2** Number of pediatric patients (0–15 years) with HCH recorded in BNDMR by year of birth, distinguishing those with at least one visit to an HCH expert in the past two years (blue) from those without such a visit (orange). Abbreviations: BNDMR, *Banque Nationale de Données Maladies Rares* [French National Registry of Rare Diseases]; HCH, hypochondroplasia*.*

**Supplementary Table 1.** Estimated regional prevalence of patients born since 2008 with achondroplasia (ACH)

| **Region** | **Number of ACH cases (N=392)*** | **Regional population born since 2008 (in millions)** | **Prevalence per 100,000** | **95% confidence interval** | **Number of confirmed cases (by genetic testing or clinically)** |
| --- | --- | --- | --- | --- | --- |
| Île-de-France | 112 | 2.6 | 4.38 | (3.51–5.11) | 108 |
| Auvergne-Rhône-Alpes | 50 | 1.6 | 3.13 | (2.26–3.99) | 49 |
| Occitanie | 23 | 1.1 | 2.09 | (1.24–2.95) | 22 |
| Normandy | 16 | 0.6 | 2.67 | (1.36–3.97) | 16 |
| Pays de la Loire | 19 | 0.8 | 2.38 | (1.31–3.44) | 16 |
| Nouvelle-Aquitaine | 30 | 1.1 | 2.73 | (1.75–3.70) | 27 |
| Provence-Alpes-Côte d'Azur | 27 | 0.9 | 3.00 | (1.87–4.13) | 24 |
| Hauts-de-France | 27 | 1.2 | 2.25 | (1.40–3.10) | 22 |
| Bourgogne-Franche-Comté | 28 | 0.5 | 5.60 | (3.53–7.67) | 26 |
| Centre-Val de Loire | 16 | 0.5 | 3.20 | (1.63–4.77) | 16 |
| Brittany | 13 | 0.6 | 2.17 | (0.99–3.34) | 11 |
| Grand Est | 14 | 1.0 | 1.40 | (0.67–2.13) | 14 |
| Overseas France | 17 | 0.6 | 2.83 | (1.49–4.18) | 15 |

*Numbers of patients by region of residence.

**Supplementary Table 2.** Estimated regional prevalence of patients born since 2008 with hypochondroplasia (HCH)

| **Region** | **Number of HCH cases (N=162)*** | **Regional population born since 2008 (in millions)** | **Prevalence per 100,000** | **95% confidence interval** | **Number of confirmed cases (by genetic testing or clinically)** |
| --- | --- | --- | --- | --- | --- |
| Île-de-France | 44 | 2.6 | 1.69 | (1.19–2.19) | 31 |
| Auvergne-Rhône-Alpes | 13 | 1.6 | 0.81 | (0.37–1.25) | 13 |
| Occitanie | 11 | 1.1 | 1.00 | (0.41–1.59) | <10 |
| Normandy | <10 | 0.6 | - | - | <10 |
| Pays de la Loire | <10 | 0.8 | - | - | <10 |
| Nouvelle-Aquitaine | 10 | 1.1 | 0.91 | (0.35–1.47) | <10 |
| Provence-Alpes-Côte d'Azur | 13 | 0.9 | 1.44 | (0.66–2.23) | <10 |
| Hauts-de-France | 11 | 1.2 | 0.92 | (0.38–1.46) | <10 |
| Bourgogne-Franche-Comté | <10 | 0.5 | - | - | <10 |
| Centre-Val de Loire | <10 | 0.5 | - | - | <10 |
| Brittany | 10 | 0.6 | 1.67 | (0.63–2.70) | <10 |
| Grand Est | 11 | 1.0 | 1.10 | (0.45–1.75) | <10 |
| Overseas France | <10 | 0.6 | - | - | <10 |

*Numbers of patients by region of residence.
